# Is Acrylamide a Hidden Threat to Muscle Health? Integrative Evidence From Epidemiological and Mechanistic Investigations

**DOI:** 10.1002/fsn3.71745

**Published:** 2026-04-10

**Authors:** Haobiao Liu, Xuefeng Yu, Qingsong Li, Yuwen Shangguan, Litao Yan, Huan Li

**Affiliations:** ^1^ Department of Occupational and Environmental Health, School of Public Health, Health Science Center Xi'an Jiaotong University Xi'an Shaanxi China; ^2^ Department of Sport and Exercise Sciences Kunsan National University Gunsan Jeollabuk‐do South Korea; ^3^ Department of Articular Orthopaedics, The First People's Hospital of Changzhou The Third Affiliated Hospital of Soochow University Changzhou Jiangsu China

**Keywords:** acrylamide, environmental contamination, inflammation, muscle mass, oxidative stress

## Abstract

Acrylamide is an environmental toxicant widely found in processed foods and tobacco smoke. While its neurotoxic and carcinogenic effects are well established, its impact on skeletal muscle health remains poorly understood. This study aimed to investigate the association between acrylamide exposure and muscle mass using epidemiological data and to explore potential mechanisms through bioinformatic analyses. We analyzed data from 1443 adults participating in the 2013–2016 National Health and Nutrition Examination Survey. Hemoglobin adducts of acrylamide (HbAA), glycidamide (HbGA), and their ratio (HbGA/HbAA) were used as biomarkers of exposure. Multivariable linear regression models were applied to examine associations with muscle mass index, with results reported as beta coefficients and 95% confidence intervals (CIs). Restricted cubic spline (RCS) models assessed dose–response relationships. Mediation analyses explored the roles of potential mediators. Network‐based bioinformatic analyses were performed to identify molecular targets and pathways. Higher HbAA levels were significantly associated with lower muscle mass index (*β* = −0.331, 95% CI: −0.543 to −0.118, *p* = 0.006), while the HbGA/HbAA ratio showed a positive association (*β* = 1.029, 95% CI: 0.702 to 1.356, *p* < 0.001). RCS further confirmed linear relationships. Mediation analyses indicated that biomarkers of inflammation and oxidative stress partially mediated these associations (mediated proportions: 5.93% to 31.39%). Bioinformatic analysis identified 346 shared genes enriched in apoptosis and inflammatory pathways. This study provides the first integrative epidemiologic and mechanistic evidence linking acrylamide exposure to reduced muscle mass. These findings highlight the potential role of inflammation and apoptosis, underscoring the importance of environmental risk factors in muscle health.

## Graphical abstract

This study provides integrative epidemiological and mechanistic evidence linking acrylamide exposure to muscle mass index. Analysis of NHANES 2013–2016 data showed that higher HbAA levels were associated with lower muscle mass index, whereas a higher HbGA/HbAA ratio was positively associated. Mediation analyses suggested partial involvement of inflammatory and oxidative stress pathways. Bioinformatic analyses further identified apoptosis‐ and inflammation‐related signaling pathways as potential mechanisms. These findings highlight a potential role of environmental toxicants in muscle health.

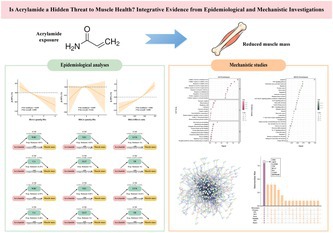

## 1 Introduction

Skeletal muscle is fundamental to human health, playing a crucial role in physical function, metabolic regulation, and overall well‐being. Declining muscle mass, particularly in aging populations, has been associated with adverse health outcomes, including frailty, falls, mobility limitations, and premature mortality (Cruz‐Jentoft et al. 2019; Cruz‐Jentoft and Sayer 2019; Veronese et al. 2019). This decline not only imposes significant burdens on individuals and healthcare systems but also represents an emerging global public health concern. While genetics, lifestyle behaviors, and nutritional status are established determinants of muscle health (Calvani et al. 2023; Cruz‐Jentoft et al. 2019), increasing attention has turned to environmental exposures as novel contributors to muscular deterioration.

In recent years, increasing attention has been paid to the contribution of environmental toxicants to musculoskeletal degeneration. A growing body of literature has linked exposure to air pollutants, heavy metals, and endocrine‐disrupting chemicals to reductions in muscle mass and function (Chiu et al. 2023; Huang et al. 2024; Liu, Chen, et al. 2025; Liu, Li, et al. 2024). However, research in this area remains in its infancy, and many potential environmental contributors have yet to be thoroughly investigated. Acrylamide—a low‐molecular‐weight, highly water‐soluble compound—is one such underexplored chemical. Despite widespread recognition of its neurotoxicity and carcinogenic potential (Delatour and Stadler 2023; Fan et al. 2023), its possible role in muscle pathophysiology has received limited scrutiny.

Acrylamide is predominantly formed during high‐temperature cooking of carbohydrate‐rich foods via the Maillard reaction (Delatour and Stadler 2023; Fan et al. 2023) and is also found in tobacco smoke and occupational settings, leading to near‐universal low‐level exposure (Esposito et al. 2022; Hagmar et al. 2001). Once absorbed, it is distributed across multiple tissues and metabolized in the liver to form glycidamide, a reactive epoxide metabolite (Sumner et al. 2003). Hemoglobin adducts of acrylamide (HbAA) and glycidamide (HbGA) serve as validated biomarkers for internal exposure in population‐based studies (Vesper et al. 2010). Biomonitoring research has consistently confirmed the presence of these adducts across diverse populations, highlighting the widespread internal burden of acrylamide (Albiach‐Delgado et al. 2022). While acrylamide has been classified as a probable human carcinogen (Group 2A) by the International Agency for Research on Cancer, experimental models have also revealed its potential to disrupt neurological, reproductive, and endocrine systems (Matoso et al. 2019; Yan et al. 2023). Notably, its implications for musculoskeletal health remain largely unexplored.

Evidence from animal studies suggests that acrylamide may exert direct myotoxic effects. Rodent models have demonstrated impaired muscle strength, altered histology, and disrupted neuromuscular transmission following acrylamide exposure, hinting at a possible link to muscle weakness or atrophy (Bai et al. 2021; Wang et al. 2021). Yet, fewer human studies have systematically examined acrylamide's relationship with muscle mass at the population level. Given the essential role of skeletal muscle in mobility, metabolic homeostasis, and resilience against chronic disease, identifying modifiable environmental risk factors is of increasing importance. Acrylamide, as a pervasive dietary and environmental contaminant, may represent a preventable contributor to muscle loss. Uncovering its association with muscle mass in human populations would not only expand the current toxicological profile of acrylamide but also offer actionable insight for public health protection. Moreover, the mechanisms by which acrylamide may influence muscle physiology are not fully elucidated. Existing evidence suggests that oxidative stress, inflammation, and cellular damage may mediate its systemic effects—processes also implicated in muscle catabolism (Huchthausen et al. 2023; Wang et al. 2022; Yan et al. 2023). Integrating molecular data with epidemiologic findings could therefore provide mechanistic insights into acrylamide‐induced muscular damage.

Against this background, the present study utilizes data from the National Health and Nutrition Examination Survey (NHANES) to investigate the association between acrylamide‐related biomarkers and skeletal muscle mass in a nationally representative adult population. In parallel, bioinformatics approaches were employed to identify potential gene targets and pathways linking acrylamide exposure to muscle‐related biological processes. Together, these complementary strategies offer a more comprehensive understanding of how environmental toxicants such as acrylamide may contribute to muscle deterioration in humans. By uncovering potential links between a ubiquitous environmental contaminant and muscle health, this study aims to inform public health policies and guide future mechanistic investigations. The findings may also provide a scientific basis for environmental interventions to preserve musculoskeletal function, particularly in vulnerable populations.

## 2 Methods

### 2.1 Study Population

**FIGURE 1:**
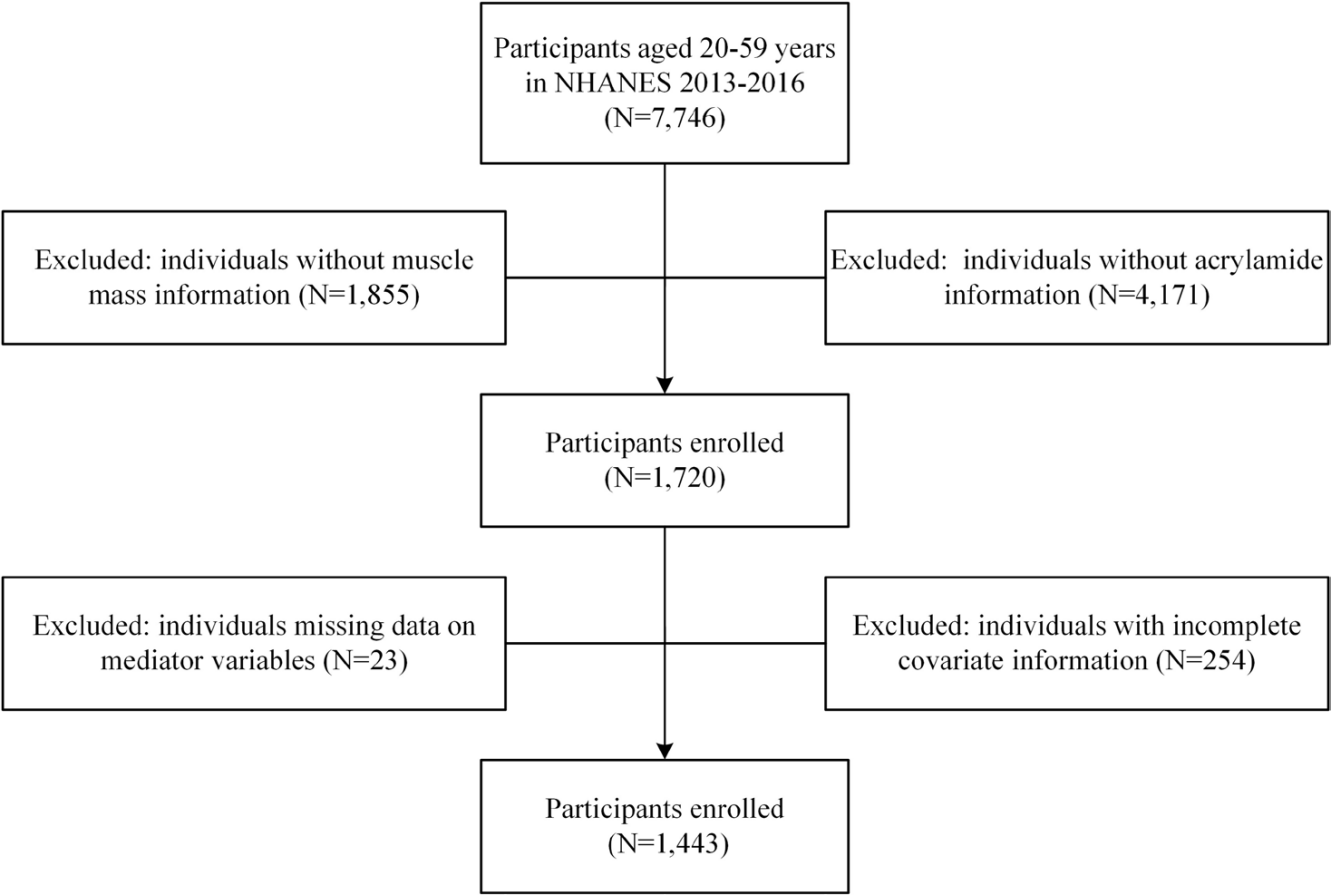
Flow diagram of the study participants included in NHANES 2013–2016.

Data for this study were derived from the NHANES 2013–2016 cycles. NHANES employs a multistage, stratified probability sampling design to obtain a representative sample of the civilian, non‐institutionalized U.S. population. Participants aged 20 years and older with available measurements of hemoglobin adducts of acrylamide and dual‐energy X‐ray absorptiometry (DXA)‐based muscle mass index were eligible for inclusion. We excluded pregnant participants and individuals with missing data on key covariates included in the fully adjusted models. After applying these inclusion and exclusion criteria, a total of 1443 participants were retained for the final analysis (Figure 1).

### 2.2 Assessment of Acrylamide Exposure

Exposure to acrylamide was evaluated using three biomarkers measured in red blood cells, namely HbAA, HbGA, and their ratio (HbGA/HbAA). Blood specimens were collected following standardized NHANES phlebotomy protocols and stored at −30°C before analysis. Quantification was performed using isotope dilution high‐performance liquid chromatography coupled with tandem mass spectrometry (HPLC‐MS/MS), utilizing a Shimadzu Prominence HPLC system coupled to an API 5000 Triple Quadrupole Mass Spectrometer (AB Sciex) equipped with a TurboIonSpray source. Detailed analytic methods, including calibration procedures and quality control protocols, are described in the publicly available NHANES laboratory methodology documentation. Due to the skewed distribution of all biomarkers, natural logarithm (ln) transformation was applied before statistical modeling to improve normality and reduce the impact of outliers. These transformed values were used as continuous exposures in subsequent regression analyses.

### 2.3 Assessment of Muscle Mass

The primary outcome was appendicular lean mass (ALM), assessed via whole‐body DXA using a Hologic Discovery A densitometer (Hologic Inc., Bedford, MA). ALM was calculated as the sum of lean soft tissue from both the upper and lower extremities. Muscle mass index was defined as ALM divided by height squared (ALM/height^2^, kg/m^2^), a widely accepted indicator of relative muscle mass. DXA is considered a reliable and widely used reference method for body composition assessment in epidemiological studies (Liu, Bao, et al. 2024; Liu, Chen, et al. 2025; Liu, Xiang, Liu, et al. 2025), providing higher accuracy than bioelectrical impedance analysis for estimating regional lean mass. NHANES applied rigorous calibration and quality control protocols throughout DXA data acquisition, and detailed scanning procedures and quality assurance protocols are available in the official NHANES documentation.

### 2.4 Covariates

We adjusted for a range of demographic, lifestyle, and clinical covariates based on prior literature and biological plausibility (Liu, Chen, et al. 2025). These included age, sex, race, education level, marital status, poverty income ratio (PIR), smoking status, drinking status, physical activity, hypertension, and diabetes. Physical activity information was obtained from the NHANES physical activity questionnaire. Weekly physical activity levels were estimated by calculating metabolic equivalent task minutes per week (MET‐min/week) based on the reported frequency and duration of moderate and vigorous recreational activities. MET values recommended by NHANES were applied, and total MET‐min/week was derived accordingly. The operational definitions and coding schemes for all covariates are provided in Table S1.

### 2.5 Mediation Analysis

To investigate whether systemic inflammation and oxidative stress biomarkers mediated the relationship between acrylamide exposure and muscle mass index, we conducted mediation analyses including white blood cell count (WBC), neutrophil count (NEU), lymphocyte count (LYM), uric acid (UA), gamma‐glutamyl transferase (GGT), and total bilirubin (TB) as potential mediators. Detailed descriptions of these biomarkers and their measurement protocols are provided in the NHANES laboratory methodology documentation (https://wwwn.cdc.gov/nchs/nhanes/Default.aspx). The mediation models decomposed the total effect of acrylamide exposure into direct and indirect components, estimating both the proportion mediated and the significance of each mediation pathway (Shen et al. 2025). Consistent with analytic practices in previous NHANES‐based mediation analyses, survey weights were not applied because formal methods for weighted mediation are not yet standardized. The analysis estimated the average causal mediation effect (ACME), average direct effect (ADE), and total effect using 1000 bootstrap replications. This approach allowed us to quantify the extent to which inflammatory and oxidative stress biomarkers might mediate the observed associations between acrylamide exposure and muscle mass index, thereby providing insights into potential biological mechanisms.

### 2.6 Bioinformatic Analysis

To elucidate the potential molecular mechanisms linking acrylamide exposure to muscle mass reduction, a network toxicology approach was employed. Genes associated with acrylamide were retrieved from the Comparative Toxicogenomics Database (CTD, https://ctdbase.org/) using the keyword “acrylamide” and those with an organism count greater than 1 were retained to ensure cross‐species relevance. The organism count filter was applied to prioritize genes supported by evidence from more than one species, thereby improving the robustness of the gene–chemical associations and reducing the likelihood of spurious single‐study annotations. In parallel, muscle mass–related genes were identified from GeneCards (https://www.genecards.org) using the search term “low muscle mass” with a relevance score threshold of > 10 to ensure biological significance. The search term was selected to specifically capture genes associated with muscle mass reduction or sarcopenia‐related phenotypes. The relevance score threshold (> 10) was applied to retain genes with stronger evidence of association in the GeneCards database, thereby reducing noise from weak or indirect annotations. The overlapping genes from both datasets were then identified as potential mediators of acrylamide‐related muscle toxicity.

Subsequent enrichment analyses were performed to investigate the biological roles of these intersected genes. Gene Ontology (GO) analysis categorized the genes into biological processes (BP), cellular components (CC), and molecular functions (MF), while Kyoto Encyclopedia of Genes and Genomes (KEGG) pathway analysis identified associated signaling pathways. Functional enrichment analyses were conducted using the R package “clusterProfiler”, with the human genome ( *Homo sapiens* ) used as the background reference gene set. *p*‐values were adjusted for multiple comparisons using the Benjamini–Hochberg false discovery rate (FDR) correction method. Pathways and GO terms with an adjusted FDR < 0.05 were considered statistically significant.

To further examine functional interactions among the overlapping genes, protein–protein interaction (PPI) networks were constructed using the STRING database (https://cn.string‐db.org/) with a minimum interaction confidence score of 0.7. The resulting PPI data were imported into Cytoscape (version 3.9.0) for topological analysis. Node importance was evaluated using eight centrality algorithms: maximal clique centrality (MCC), maximum neighborhood component (MNC), edge percolated component (EPC), degree, betweenness, closeness, stress, and radiality. The top 20 genes ranked by each metric were cross‐referenced to identify hub genes that may play key roles in the regulatory network.

### 2.7 Statistical Analysis

In the present study, analyses accounted for the complex multistage sampling design of NHANES by incorporating appropriate sampling weights, strata, and primary sampling units, in accordance with NHANES analytical guidelines and previous studies (NCHS 2024; Liu and Chen 2025, 2026). Specifically, we used the laboratory subsample weight (WTSA2YR) as the exposure variable (hemoglobin adducts) was measured in the corresponding laboratory subsample.

Descriptive statistics were presented as means with standard errors (SE) for continuous variables and as frequencies with percentages for categorical variables. Survey‐weighted linear regression models were used to assess the associations between acrylamide biomarkers and muscle mass index, with beta coefficients and 95% confidence intervals (CIs) reported. Three models were constructed: the crude model, which did not account for any covariates; the partially adjusted model, which controlled for age, sex, and race; and the fully adjusted model, which additionally incorporated socioeconomic factors, lifestyle variables, and clinical parameters. To assess potential dose–response relationships, survey‐weighted restricted cubic spline (RCS) models were fitted with knots placed at the 10th, 50th, and 90th percentiles, using the median as the reference, to allow flexible modeling of nonlinear associations (Liu, Xiang, and Chen 2025; Liu et al. 2026).

Subgroup analyses were conducted to examine potential effect modification, with interaction terms used to test statistical heterogeneity. To ensure the robustness of findings, several sensitivity analyses were performed: (1) multiple imputation for missing covariates; (2) exclusion of individuals with a history of cancer; (3) exclusion of outliers with biomarker levels exceeding three standard deviations from the mean; and (4) reanalysis using unweighted models that did not account for NHANES's complex sampling design.

All analyses were conducted using R software (version 4.4.0), and statistical significance was defined as a two‐sided *p*‐value < 0.05.

## 3 Results

### 3.1 Participant Characteristics

A total of 1443 participants were included in the final analysis, with a mean age of 39.34 years. Females accounted for 49.11% of the study sample. The majority of participants were Non‐Hispanic White (63.37%), and 66.68% had attained a college degree or higher. A comprehensive summary of baseline demographic, socioeconomic, lifestyle, and clinical characteristics is presented in Table 1. Participant characteristics stratified by quartiles of HbAA, HbGA, and HbGA/HbAA ratio are provided in Tables S2–S4.

**TABLE 1:** Weighted characteristics of the study participants.

Characteristic	Total (*N* = 1443)
Age, years	39.34 (11.74)
Sex	
Male	727 (50.89)
Female	716 (49.11)
Race	
Non‐Hispanic White	553 (63.37)
Non‐Hispanic Black	275 (11.06)
Hispanic	383 (16.65)
Other	232 (8.92)
Educational attainment	
Less than high school	263 (12.87)
High school graduate or equivalent	308 (20.45)
Some college or associated degree	465 (34.43)
College graduate or above	407 (32.25)
Marital status	
Single	567 (37.45)
Couple	876 (62.55)
Poverty income ratio	
< 1.0	291 (14.51)
1.0–3.0	596 (36.27)
> 3.0	556 (49.22)
Smoking status	
Non‐smoker	850 (58.83)
Former smoker	243 (18.45)
Current smoker	350 (22.72)
Drinking status	
Non‐drinker	365 (19.85)
Low to moderate	961 (70.31)
Heavy	117 (9.84)
Physical activity	
Inactive	860 (56.95)
Active	583 (43.05)
Hypertension	
No	1055 (73.93)
Yes	388 (26.07)
Diabetes	
No	1310 (92.62)
Yes	133 (7.38)

*Note:* The numbers of participants in each category are unweighted observed frequencies, while means, standard errors, and percentages are population‐weighted.

### 3.2 Associations Between Acrylamide Biomarkers and Muscle Mass Index

The associations between acrylamide‐related biomarkers and muscle mass index are summarized in Table 2. In the crude model, a one‐unit increase in ln‐transformed HbAA was significantly associated with lower muscle mass index (*β* = −0.178, 95% CI: −0.349 to −0.008, *p* = 0.041), while the ln‐transformed HbGA/HbAA ratio exhibited a positive correlation (*β* = 0.395, 95% CI: 0.068–0.722, *p* = 0.020). These associations remained consistent in the partially adjusted model, with HbAA negatively associated with muscle mass index (*β* = −0.318, 95% CI: −0.495 to −0.141, *p* = 0.001), and the HbGA/HbAA ratio exhibiting a strengthened positive association (*β* = 1.075, 95% CI: 0.798–1.352, *p* < 0.001). In the fully adjusted model, which accounted for demographic, socioeconomic, lifestyle, and clinical covariates, both associations persisted. Specifically, each one‐unit increase in ln‐transformed HbAA remained inversely associated with muscle mass index (*β* = −0.331, 95% CI: −0.543 to −0.118, *p* = 0.006), whereas the HbGA/HbAA ratio continued to be positively associated (*β* = 1.029, 95% CI: 0.702–1.356, *p* < 0.001).

**TABLE 2:** Association between acrylamide exposure and muscle mass index among U.S. adults in NHANES 2013–2016.

Variable	Crude model		Partially adjusted model		Fully adjusted model	
	*β* (95% CI)	*p*	*β* (95% CI)	*p*	*β* (95% CI)	*p*
HbAA						
Per unit increase	**−0.178 (−0.349, −0.008)**	**0.041**	**−0.318 (−0.495, −0.141)**	**0.001**	**−0.331 (−0.543, −0.118)**	**0.006**
Quartile 1	Reference		Reference		Reference	
Quartile 2	−0.313 (−0.655, 0.029)	0.071	−0.252 (−0.589, 0.085)	0.136	−0.266 (−0.653, 0.121)	0.155
Quartile 3	−0.197 (−0.461, 0.066)	0.136	−0.230 (−0.493, 0.033)	0.083	−0.226 (−0.499, 0.047)	0.094
Quartile 4	**−0.350 (−0.655, −0.045)**	**0.026**	**−0.487 (−0.795, −0.179)**	**0.003**	**−0.445 (−0.831, −0.059)**	**0.028**
HbGA						
Per unit increase	−0.099 (−0.314, 0.115)	0.350	−0.081 (−0.311, 0.148)	0.472	0.016 (−0.215, 0.246)	0.883
Quartile 1	Reference		Reference		Reference	
Quartile 2	−0.038 (−0.318, 0.241)	0.780	0.064 (−0.147, 0.275)	0.538	0.050 (−0.163, 0.262)	0.610
Quartile 3	0.041 (−0.237, 0.319)	0.765	0.084 (−0.151, 0.319)	0.467	0.081 (−0.179, 0.341)	0.498
Quartile 4	−0.162 (−0.492, 0.169)	0.324	−0.072 (−0.397, 0.253)	0.651	0.097 (−0.254, 0.447)	0.548
HbGA/HbAA ratio						
Per unit increase	**0.395 (0.068, 0.722)**	**0.020**	**1.075 (0.798, 1.352)**	**< 0.001**	**1.029 (0.702, 1.356)**	**< 0.001**
Quartile 1	Reference		Reference		Reference	
Quartile 2	−0.055 (−0.392, 0.282)	0.739	0.175 (−0.029, 0.378)	0.089	0.183 (−0.076, 0.443)	0.144
Quartile 3	0.071 (−0.301, 0.443)	0.698	**0.400 (0.106, 0.695)**	**0.010**	**0.364 (0.017, 0.711)**	**0.042**
Quartile 4	0.290 (−0.039, 0.620)	0.082	**0.858 (0.617, 1.099)**	**< 0.001**	**0.800 (0.507, 1.092)**	**< 0.001**

*Note:* Crude model, no covariate was adjusted; Partially adjusted model, adjusted for age, sex, race; Fully adjusted model, adjusted for age, sex, race, educational level, marital status, poverty income ratio, smoking status, drinking status, physical activity, hypertension, and diabetes. Blood acrylamide was natural logarithm transformed before analyses. Results in bold indicate statistical significance.

Abbreviations: CI, confidence interval; HbAA, hemoglobin adducts of acrylamide; HbGA, hemoglobin adducts of glycidamide.

When exposure biomarkers were categorized into quartiles, participants in the highest quartile of HbAA exhibited significantly lower muscle mass index compared to the lowest quartile (*β* = −0.445, 95% CI: −0.831 to −0.059, *p* = 0.028). Conversely, higher HbGA/HbAA ratios were associated with increased muscle mass index in both the third (*β* = 0.364, 95% CI: 0.017–0.711, *p* = 0.042) and fourth (*β* = 0.800, 95% CI: 0.507–1.092, *p* < 0.001) quartiles. No statistically significant relationship was found between HbGA levels and muscle mass index in any model.

**FIGURE 2:**
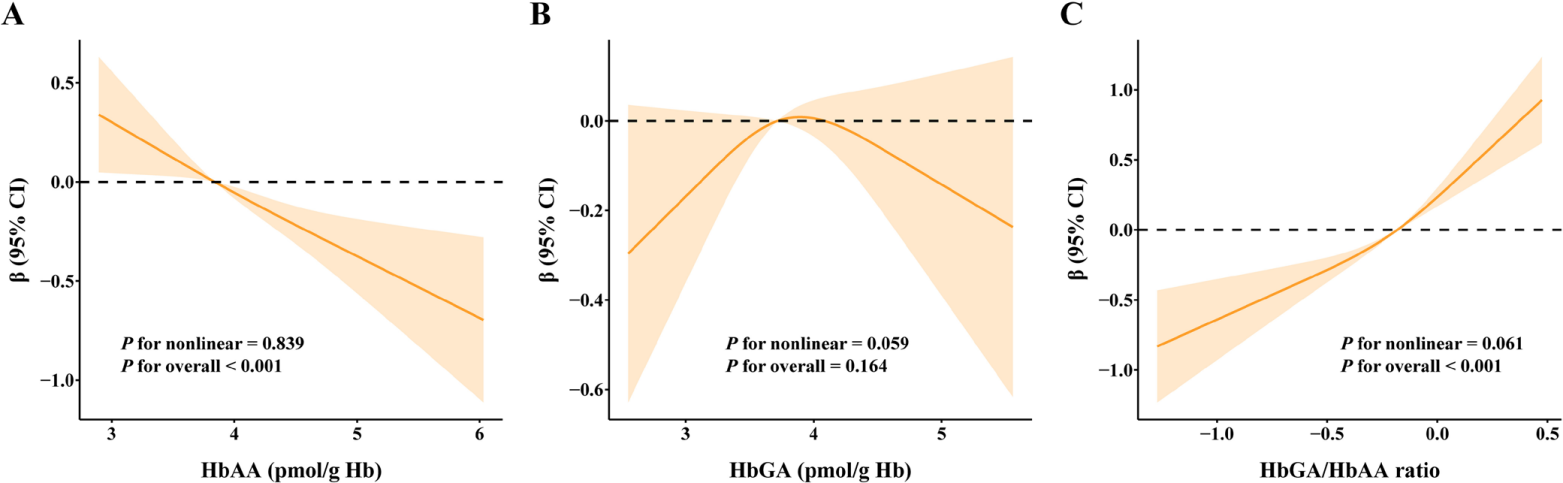
Restricted cubic spline analysis between acrylamide and muscle mass index among U.S. adults in NHANES 2013–2016. (A) HbAA. (B) HbGA. (C) HbGA/HbAA ratio. The models were adjusted for age, sex, race, educational level, marital status, poverty income ratio, smoking status, drinking status, physical activity, hypertension, and diabetes. The solid line represents an estimate of the beta coefficient, and the shaded area represents the 95% CI. Blood acrylamide was natural logarithm transformed before analyses. CI, confidence interval; HbAA, hemoglobin adducts of acrylamide; HbGA, hemoglobin adducts of glycidamide.

These findings were further supported by RCS analyses (Figure 2), which revealed a dose‐dependent decline in muscle mass index with increasing HbAA and a corresponding increase with higher HbGA/HbAA ratios. The spline curves demonstrated linear trends without significant evidence of non‐linearity (*p =* 0.839 for HbAA and *p* = 0.061 for HbGA/HbAA ratio).

### 3.3 Mediation by Systemic Inflammation and Oxidative Stress

**FIGURE 3:**
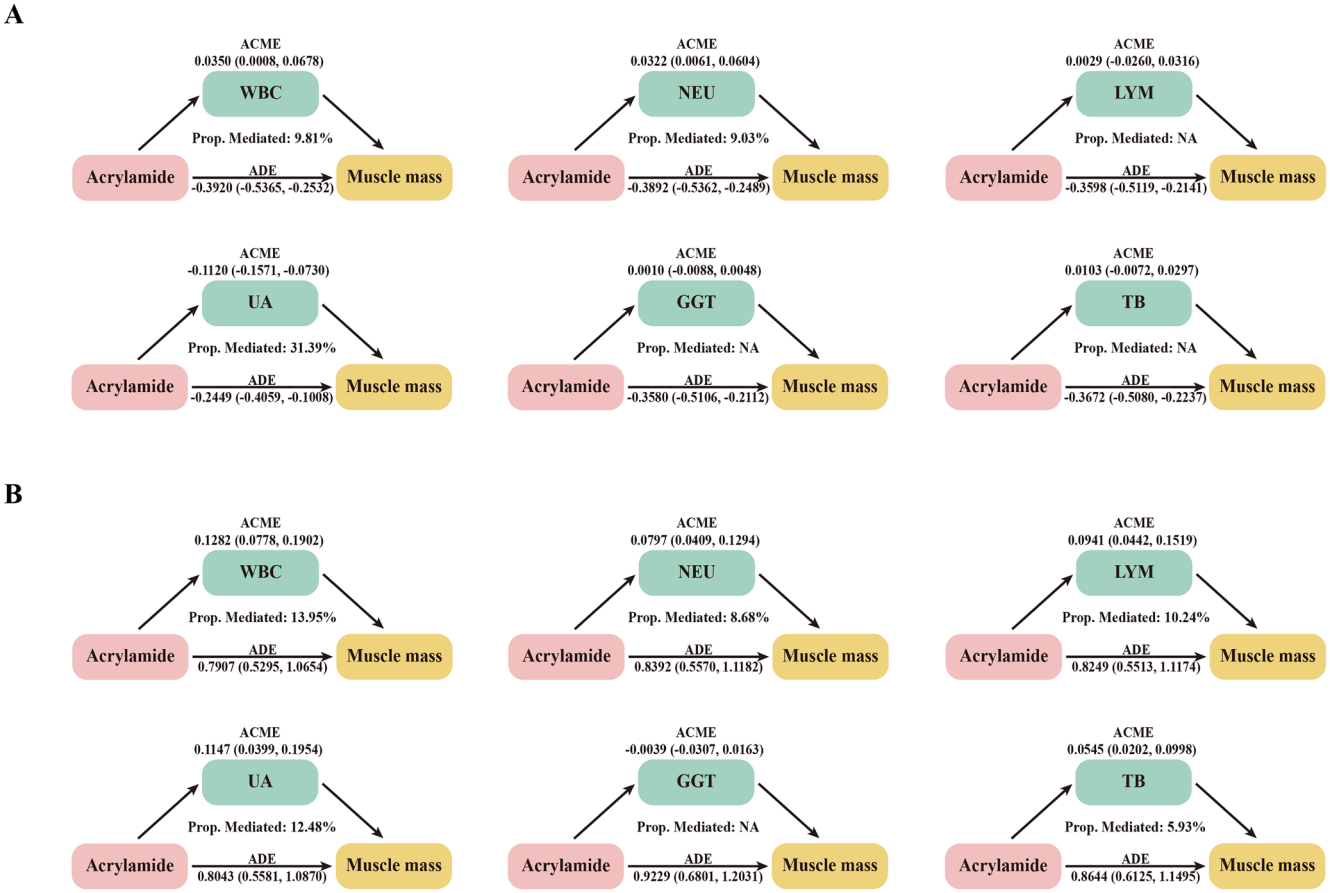
Mediation effects of inflammation and oxidant stress in the association of acrylamide with muscle mass index among U.S. adults in NHANES 2013–2016. (A) HbAA. (B) HbGA/HbAA ratio. The models were adjusted for age, sex, race, educational level, marital status, poverty income ratio, smoking status, drinking status, physical activity, hypertension, and diabetes. Blood acrylamide was natural logarithm transformed before analyses. ACME, average causal mediation effects; ADE, average direct effects; GGT, gamma‐glutamyl transferase; HbAA, hemoglobin adducts of acrylamide; HbGA, hemoglobin adducts of glycidamide; LYM, lymphocyte; NEU, neutrophil; TB, total bilirubin; UA, uric acid; WBC, white blood cell.

To investigate the underlying biological mechanisms linking acrylamide exposure to reduced muscle mass, mediation analyses were performed using selected biomarkers of systemic inflammation and oxidative stress. For HbAA, significant indirect effects were identified through WBC, NEU, and UA. Specifically, WBC mediated 9.81% of the total association between HbAA and muscle mass index, NEU accounted for 9.03%, and UA contributed the largest proportion at 31.39% (Figure 3A). These results suggest that both inflammatory and oxidative processes may partially mediate the deleterious effects of acrylamide exposure on skeletal muscle.

In the analysis of the HbGA/HbAA ratio, five biomarkers were identified as significant mediators. Specifically, WBC, NEU, LYM, UA, and TB mediated 13.95%, 8.68%, 10.24%, 12.48%, and 5.93% of the positive association between the metabolic ratio and muscle mass index, respectively (Figure 3B). These findings suggest that although a higher HbGA/HbAA ratio is associated with modest increases in systemic inflammation and oxidative stress markers, the overall effect on muscle mass index remains beneficial. This may reflect more efficient metabolic conversion of acrylamide into glycidamide, potentially reducing the accumulation of harmful intermediates and mitigating adverse effects on muscle health.

No significant mediation effect was observed for GGT in either model (Table S5). Collectively, these results support the involvement of immune‐inflammatory and redox‐sensitive pathways in the toxicological profile of acrylamide, providing mechanistic insight into the observed epidemiologic associations.

### 3.4 Subgroup Analyses

To further examine the consistency and potential effect modification of the observed associations, stratified analyses were conducted for HbAA and HbGA/HbAA ratio across subgroups. For HbAA, the inverse association with muscle mass index remained generally stable across all examined subgroups. No significant interaction effects were observed (all *P* for interaction > 0.05), suggesting that the detrimental association between acrylamide exposure and reduced muscle mass index was consistent irrespective of baseline characteristics (Table S6). In contrast, for the HbGA/HbAA ratio, a significant interaction was detected with physical activity status (*P* for interaction = 0.028). The positive association between HbGA/HbAA ratio and muscle mass index appeared to be more pronounced among individuals with low physical activity levels. Specifically, among physically inactive participants, a higher HbGA/HbAA ratio was associated with increased muscle mass index (*β* = 1.311, 95% CI: 0.982–1.641), whereas the association was attenuated in the physically active group (Table S7). This finding suggests that metabolic disposition toward glycidamide formation may play a more prominent role in muscle maintenance among those with insufficient physical activity. No other significant interactions were observed for the HbGA/HbAA ratio in stratified models. These results highlight a potential moderating effect of physical activity on the relationship between acrylamide metabolism and muscle mass index.

### 3.5 Potential Targets and Mechanisms Underlying Acrylamide Exposure and Muscle Mass

A total of 884 acrylamide‐related genes were identified from the CTD based on an organism count > 1, while 4488 muscle mass‐associated genes were retrieved from GeneCards using a relevance score > 10. By intersecting these two gene sets, 346 common targets were obtained, suggesting potential molecular links between acrylamide exposure and muscle mass regulation (Figure S1).

**FIGURE 4:**
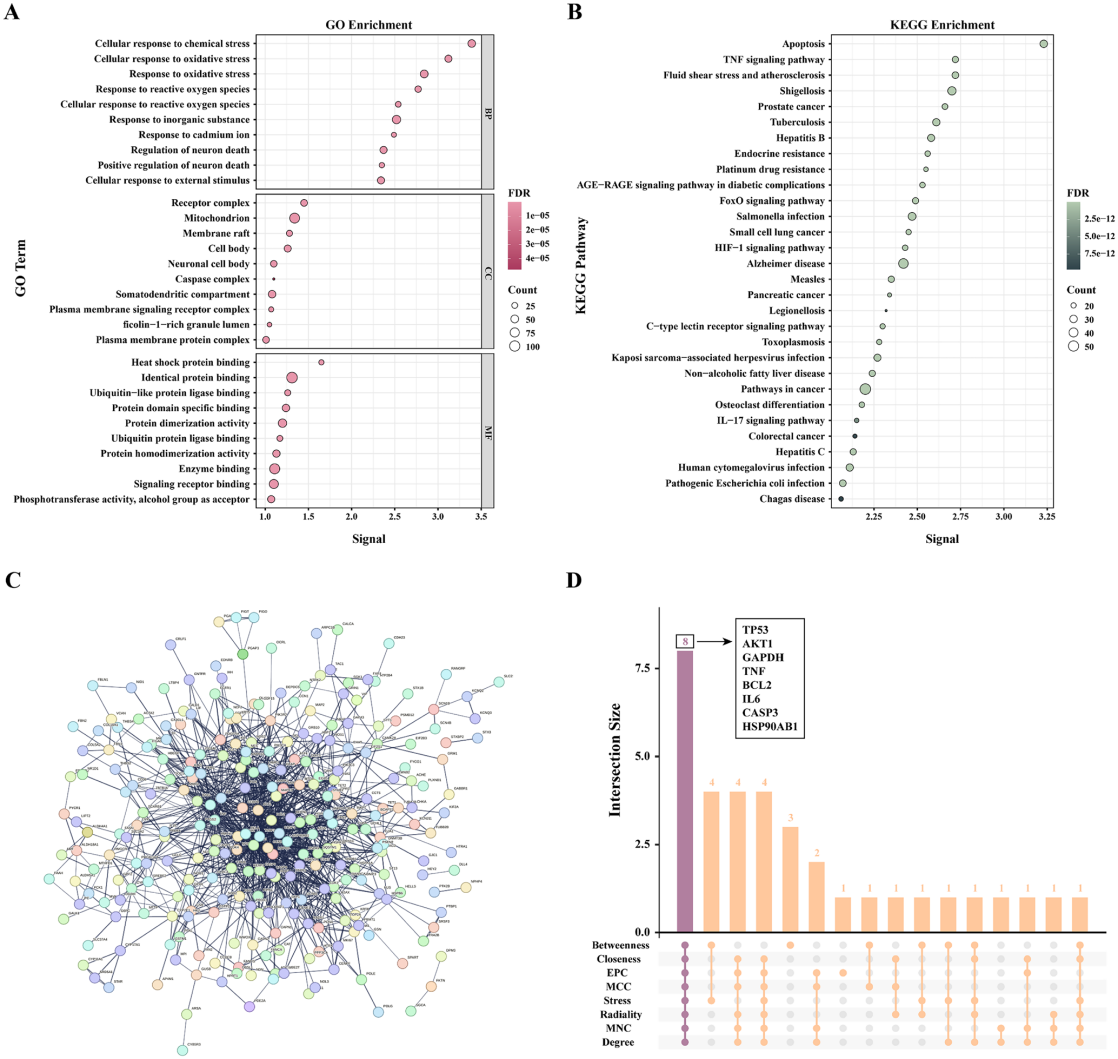
Bioinformatic analysis of potential targets and mechanisms linking acrylamide exposure to reduced muscle mass. (A) GO analysis; (B) KEGG analysis; (C) PPI network of overlapping targets; (D) Venn graph of intersection targets utilized topological algorithms via Cytoscape. BP, biological process; CC, cellular component; GO, Gene Ontology; KEGG, Kyoto Encyclopedia of Genes and Genomes; MF, molecular function; PPI, protein–protein interaction.

Functional enrichment analysis (Figure 4A) of the overlapping targets revealed significant biological involvement in apoptosis‐related processes. GO terms were enriched in biological processes such as “Cellular response to chemical stress”, “Cellular response to oxidative stress”, and “Response to oxidative stress”. Cellular component enrichment was mainly associated with the “Receptor complex”, “Mitochondrion”, and “Membrane raft”, while molecular function terms highlighted “Heat shock protein binding”, “Identical protein binding”, and “Ubiquitin‐like protein ligase binding”. KEGG pathway analysis (Figure 4B) further underscored the involvement of apoptosis and inflammation‐related signaling cascades. Prominent pathways included the “Apoptosis”, “TNF signaling pathway”, “FoxO signaling pathway”, “HIF‐1 signaling pathway”, and “IL‐17 signaling pathway”, indicating that acrylamide may affect muscle integrity through stress, inflammatory, and cell death mechanisms.

Protein–protein interaction (PPI) network analysis (Figure 4C) of the 346 intersected genes was constructed using the STRING database with a confidence score threshold of > 0.7 and visualized in Cytoscape. Topological analysis across eight algorithms (MCC, MNC, EPC, degree, betweenness, closeness, stress, and radiality) consistently identified TP53, AKT1, GAPDH, TNF, BCL2, IL6, CASP3, and HSP90AB1 as central hub genes (Figure 4D). These proteins are well‐recognized regulators of apoptosis, inflammatory responses, and cellular homeostasis, further supporting the hypothesis that acrylamide exposure may impair muscle health through apoptotic and stress‐related molecular pathways.

### 3.6 Sensitivity Analyses

All sensitivity analyses yielded results consistent with those of the primary models, supporting the robustness of the observed associations. After multiple imputations for missing covariates, the direction and magnitude of the associations between acrylamide‐related biomarkers and muscle mass remained stable, with negligible changes in effect estimates or confidence intervals (Table S8). Exclusion of participants with a self‐reported history of cancer did not materially alter the associations, suggesting that the observed relationships were not confounded by underlying malignancies or cancer‐related muscle wasting (Table S9). The exclusion of individuals with extreme exposure values also did not meaningfully change the results, indicating that the associations were not driven by outliers (Table S10). Finally, the unweighted analyses produced similar estimates to those obtained using the NHANES sampling weights, reinforcing that the observed relationships were not substantially influenced by the complex survey design (Table S11). Collectively, these findings confirm the robustness and stability of the associations between acrylamide exposure biomarkers and muscle mass index across a variety of analytical conditions.

## 4 Discussion

To the best of our knowledge, this is the first study to integratively apply both epidemiological analysis and bioinformatic methods to investigate the potential relationship between internal acrylamide exposure and skeletal muscle mass. Utilizing a nationally representative dataset from NHANES alongside network toxicology analysis, we observed a significant inverse association between HbAA and muscle mass index, whereas the HbGA/HbAA ratio showed a positive association. These significant relationships were further supported by RCS modeling and remained robust across subgroups and multiple sensitivity analyses. Future studies with wider exposure ranges, particularly among occupationally exposed populations, may help clarify whether threshold effects or nonlinear dose–response relationships emerge at higher levels of acrylamide exposure. Furthermore, bioinformatics analysis revealed that the overlapping molecular targets between acrylamide and muscle mass were significantly enriched in apoptotic and inflammatory pathways, offering novel mechanistic insights into how acrylamide may impact muscle physiology.

Acrylamide is a ubiquitous environmental contaminant formed during high‐temperature cooking of carbohydrate‐rich foods and also found in tobacco smoke. Although it has been extensively studied for its neurotoxicity, carcinogenicity, and reproductive toxicity, its impact on skeletal muscle health has remained largely unexplored. However, emerging experimental studies have begun to report adverse effects of acrylamide on the musculoskeletal system, including evidence of skeletal tissue damage and increased oxidative stress following chronic exposure in animal models (Safwat et al. 2022; Zhang et al. 2024). The present study not only expands the toxicological profile of acrylamide but also underscores the need to consider environmental exposures in the context of musculoskeletal decline and sarcopenia. Unlike prior studies that focused on single indicators, our findings differentiate between the effects of HbAA, HbGA, and the HbGA/HbAA ratio. HbAA showed a consistent negative association with muscle mass index, whereas HbGA was not significantly associated. Interestingly, a higher HbGA/HbAA ratio, indicative of more efficient acrylamide metabolism, was positively associated with muscle mass index. This relationship may reflect inter‐individual variability in acrylamide metabolism rather than a direct protective effect of glycidamide. This ratio has been proposed as a proxy for metabolic conversion efficiency, potentially capturing differences in cytochrome P450‐mediated bioactivation and downstream detoxification pathways. Individuals with a higher ratio may therefore exhibit more effective metabolic processing of acrylamide. Thus, the metabolic ratio may represent a toxicokinetically relevant indicator of internal exposure, especially in the context of chronic outcomes such as muscle loss. Nevertheless, the biological interpretation of this biomarker remains complex, and further studies integrating metabolomic profiling or experimental toxicology approaches are warranted to clarify the metabolic mechanisms underlying this association. This research provides novel evidence linking environmental exposure to acrylamide with a clinical phenotype of public health importance. Historically, studies on sarcopenia have focused on aging, nutrition, and physical activity. However, emerging data suggest that environmental pollutants may also play a critical role. Our findings support this paradigm shift by identifying acrylamide as a previously underrecognized risk factor for reduced muscle mass.

The biological plausibility of our findings is further supported by the mediation analysis, which demonstrated that systemic inflammation and oxidative stress markers—such as WBC, NEU, and UA—partially mediated the association between HbAA and muscle mass index. For the HbGA/HbAA ratio, mediation was also observed for LYM and TB, highlighting both immune and redox pathways. These results align with existing literature suggesting that chronic low‐grade inflammation and oxidative stress are key mechanisms in muscle protein catabolism (Chernyavskij et al. 2023; Deger et al. 2017). Recent experimental research further supports the role of oxidative stress and inflammatory signaling in skeletal muscle dysfunction, as excessive reactive oxygen species and chronic inflammatory activation can disrupt muscle protein homeostasis and accelerate muscle degeneration (Chen et al. 2022). Nevertheless, it should be noted that the identified mediators explained only a modest portion of the observed associations. Potential mechanisms such as insulin resistance, mitochondrial dysfunction, endocrine regulation, or muscle‐specific signaling pathways may also contribute to the relationship and warrant further investigation in future mechanistic studies. In addition, the mediation analyses were conducted without applying survey weights due to methodological constraints, and therefore the estimated mediation effects should be interpreted cautiously.

Our bioinformatics analysis identified 346 overlapping genes linking acrylamide exposure and muscle mass. Functional enrichment revealed prominent roles for apoptosis‐related pathways, including TNF, HIF‐1, IL‐17, and FoxO signaling pathways. Among the eight hub genes—TP53, AKT1, GAPDH, TNF, BCL2, IL6, CASP3, and HSP90AB1—many are canonical regulators of apoptosis, cell survival, and inflammation. These genes are known to influence muscle differentiation, repair, and atrophy. For instance, TP53 and CASP3 are central to apoptosis, while AKT1 is critical for muscle hypertrophy and regeneration (Voskarides and Giannopoulou 2023; Wu et al. 2017). TNF and IL6 promote muscle wasting through pro‐inflammatory signaling (Webster et al. 2020). The anti‐apoptotic gene BCL2 and molecular chaperone HSP90AB1 further highlight potential disruptions in cellular homeostasis and proteostasis (Haase and Fitze 2016; Singh et al. 2019). Taken together, these findings suggest that acrylamide may contribute to muscle degradation not through isolated molecular actions but by orchestrating a network of apoptotic, oxidative, and inflammatory events. Supporting this interpretation, toxicological studies have demonstrated that acrylamide exposure induces oxidative stress, inflammatory responses, and apoptosis across multiple tissues, highlighting these pathways as central mechanisms underlying acrylamide toxicity (Alqahtani et al. 2024; Deng et al. 2022). This network‐based toxicity paradigm is consistent with contemporary views in systems toxicology and reinforces the notion that environmental pollutants often exert multifaceted biological effects. Nevertheless, it should be emphasized that although the network toxicology analysis highlighted several hub genes and enriched pathways related to apoptosis and inflammatory signaling, these findings should be interpreted primarily as hypothesis‐generating rather than definitive mechanistic evidence. Experimental studies using muscle cell models or animal systems will be necessary to determine whether acrylamide exposure directly alters the expression or activity of these candidate targets.

Despite several strengths, this study also has limitations that should be acknowledged. First, the cross‐sectional design of the NHANES dataset limits the ability to infer causality. Because acrylamide exposure biomarkers and muscle mass were assessed at the same time point, reverse causation and residual confounding cannot be completely excluded. Future longitudinal cohort studies and experimental investigations are needed to clarify the temporal relationship and causal mechanisms linking acrylamide exposure with skeletal muscle decline. Second, the biomarkers used in this study primarily reflect recent internal exposure over the approximate lifespan of erythrocytes. Therefore, they may not fully capture long‐term cumulative exposure resulting from habitual dietary intake. Future studies incorporating repeated biomarker measurements or detailed dietary assessments may provide a more comprehensive evaluation of chronic acrylamide exposure. Third, although NHANES employs a nationally representative sampling framework, the relatively modest sample size may limit statistical power for stratified analyses across demographic subgroups such as age, sex, smoking status, or ethnicity. Additionally, because NHANES represents the U.S. population, caution is warranted when generalizing these findings to other regions with different dietary habits or sources of acrylamide exposure. Fourth, since the bioinformatic findings were derived from curated databases rather than direct experimental evidence, validation of these pathways using muscle‐specific gene expression data is necessary. Lastly, our outcome focused on ALM/height^2^, which captures mass but not strength or function, key components in the clinical definition of sarcopenia. Future research incorporating grip strength, gait speed, or muscle quality indices could offer a more comprehensive picture.

From a food and nutrition perspective, dietary acrylamide exposure in the general population mainly results from high‐temperature processing of carbohydrate‐rich foods. Thermal cooking processes such as frying, baking, and roasting can promote acrylamide formation through the Maillard reaction between asparagine and reducing sugars. Strategies that reduce acrylamide formation during food processing, including optimization of cooking conditions and ingredient modification, may therefore help lower dietary exposure. In addition, nutrition guidance promoting balanced dietary patterns and reduced consumption of heavily processed foods may help minimize long‐term acrylamide intake.

## 5 Conclusion

In conclusion, this integrative investigation combining population epidemiology with network toxicology analysis provides compelling evidence that internal exposure to acrylamide, particularly higher levels of HbAA, may negatively affect skeletal muscle mass index. Conversely, a higher HbGA/HbAA ratio, reflecting more efficient metabolic conversion, was positively associated with muscle mass index, suggesting potential metabolic modulation. The association appears to be partly mediated by systemic inflammation and oxidative stress, and enrichment analysis highlighted molecular pathways related to apoptosis, inflammation, and oxidative damage. These findings unveil a previously unrecognized dimension of acrylamide toxicity and underscore the potential of environmental toxicants in contributing to muscle loss. As the first study to integrate epidemiological data with mechanistic bioinformatics on this topic, our results call for further longitudinal and experimental investigations. Addressing modifiable environmental contributors to muscle decline may be critical for preventing frailty and disability, especially in aging societies.

## Author Contributions

Haobiao Liu: conceptualization, methodology, formal analysis, visualization, writing – original draft preparation. Xuefeng Yu: data curation, validation, writing – review and editing. Qingsong Li: data curation, visualization, writing – review and editing. Yuwen Shangguan: supervision, writing – review and editing. Litao Yan: writing – review and editing. Huan Li: funding acquisition, writing – review and editing. All authors have read and agreed to the published version of the manuscript.

## Funding

This research was supported by the Science and Technology Project of Changzhou Municipal Health Commission (QY202502) and Applied Basic Research Project of Changzhou (CJ20252030). The funding bodies had no roles in the design of the study and collection, analysis, and interpretation of data and in writing the manuscript.

## Ethics Statement

This research is based on an analysis of publicly available data from NHANES, obtained with approval from the Ethics Review Board of the National Center for Health Statistics (https://www.cdc.gov/nchs/nhanes/about/erb.html?CDC_AAref_Val=https://www.cdc.gov/nchs/nhanes/irba98.htm). Since the NHANES public use datasets do not include any personally identifiable information, the requirement for ethical review and approval was waived for this study. All procedures were performed in compliance with the World Medical Association Declaration of Helsinki.

## Consent

Informed consent was obtained from all subjects involved in the study.

## Conflicts of Interest

The authors declare no conflicts of interest.

## Supporting information


**Table S1:** Category and definition of covariates.
**Table S2:** Basic characteristics of participants based on quartiles of HbAA.
**Table S3:** Basic characteristics of participants based on quartiles of HbGA.
**Table S4:** Basic characteristics of participants based on quartiles of HbGA/HbAA ratio.
**Table S5:** Mediation effects of inflammation and oxidant stress in the association of acrylamide with muscle mass index among U.S. adults in NHANES 2013–2016.
**Table S6:** Stratified analysis of the weighted association between HbAA and muscle mass index among U.S. adults in NHANES 2013–2016.
**Table S7:** Stratified analysis of the weighted association between HbGA/HbAA ratio and muscle mass index among U.S. adults in NHANES 2013–2016.
**Table S8:** Association between acrylamide and muscle mass index among U.S. adults in NHANES 2013–2016, after applying multiple imputations for missing covariates.
**Table S9:** Association between acrylamide and muscle mass index among U.S. adults in NHANES 2013–2016, after excluding those who had a history of cancer.
**Table S10:** Association between acrylamide and muscle mass index among U.S. adults in NHANES 2013–2016, after excluding participants identified as outliers in their exposure values.
**Table S11:** Association between acrylamide and muscle mass index among U.S. adults in NHANES 2013–2016, using unweighted data.
**Figure S1:** Venn graph of potential targets of acrylamide exposure and reduced muscle mass.

## Data Availability

The data utilized in this research can be accessed publicly through the National Health and Nutrition Examination Survey website (https://wwwn.cdc.gov/nchs/nhanes/Default.aspx).
