# Supplementary material for: Is Acrylamide a Hidden Threat to Muscle Health? Integrative Evidence From Epidemiological and Mechanistic Investigations
Source: Food Sci Nutr. 2026 Apr 10;14(4):e71745. doi: 10.1002/fsn3.71745 (PMC13067203; doi:10.1002/fsn3.71745)
Supplement: Supplementary file 1 — Table S1: Category and definition of covariates. Table S2: Basic characteristics of participants based on quartiles of HbAA. Table S3: Basic characteristics of participants based on quartiles of HbGA. Table S4: Basic characteristics of participants based on quartiles of HbGA/HbAA ratio. Table S5: Mediation effects of inflammation and oxidant stress in the association of acrylamide with muscle mass index among U.S. adults in NHANES 2013–2016. Table S6: Stratified analysis of the weighted association between HbAA and muscle mass index among U.S. adults in NHANES 2013–2016. Table S7: Stratified analysis of the weighted association between HbGA/HbAA ratio and muscle mass index among U.S. adults in NHANES 2013–2016. Table S8: Association between acrylamide and muscle mass index among U.S. adults in NHANES 2013–2016, after applying multiple imputations for missing covariates. Table S9: Association between acrylamide and muscle mass index among U.S. adults in NHANES 2013–2016, after excluding those who had a history of cancer. Table S10: Association between acrylamide and muscle mass index among U.S. adults in NHANES 2013–2016, after excluding participants identified as outliers in their exposure values. Table S11: Association between acrylamide and muscle mass index among U.S. adults in NHANES 2013–2016, using unweighted data. Figure S1: Venn graph of potential targets of acrylamide exposure and reduced muscle mass. [file FSN3-14-e71745-s001.docx]

**Supplementary Material**

**Table S1.** Category and definition of covariates.

**Table S2.** Basic characteristics of participants based on quartiles of HbAA.

**Table S3.** Basic characteristics of participants based on quartiles of HbGA.

**Table S4.** Basic characteristics of participants based on quartiles of HbGA/HbAA ratio.

**Table S5.** Mediation effects of inflammation and oxidant stress in the association of acrylamide with muscle mass index among U.S. adults in NHANES 2013-2016.

**Table S6.** Stratified analysis of the weighted association between HbAA and muscle mass index among U.S. adults in NHANES 2013-2016.

**Table S7.** Stratified analysis of the weighted association between HbGA/HbAA ratio and muscle mass index among U.S. adults in NHANES 2013-2016.

**Table S8.** Association between acrylamide and muscle mass index among U.S. adults in NHANES 2013-2016, after applying multiple imputations for missing covariates.

**Table S9.** Association between acrylamide and muscle mass index among U.S. adults in NHANES 2013-2016, after excluding those who had a history of cancer.

**Table S10.** Association between acrylamide and muscle mass index among U.S. adults in NHANES 2013-2016, after excluding participants identified as outliers in their exposure values.

**Table S11.** Association between acrylamide and muscle mass index among U.S. adults in NHANES 2013-2016, using unweighted data.

**Figure S1.** Venn graph of potential targets of acrylamide exposure and reduced muscle mass.

**Table S1.** Category and definition of covariates.

| **Variable** | **Category and definition** |
| --- | --- |
| Age | Continuous variable. |
| Sex | Male, Female. |
| Race | Non-Hispanic White, Non-Hispanic Black, Hispanic, and Other. |
| Educational attainment | Less than high school, High school graduate or equivalent, Some college or associated degree, and College graduate or above. |
| Marital status | Single: Never married, Widowed, Divorced, or Separated.  Couple: Married or Living with a partner. |
| Poverty income ratio | <1.0, 1.0-3.0, and >3.0. |
| Smoking status | Non-smokers: reporting smoking less than 100 cigarettes in their lifetime.  Former smokers: smoking more than 100 cigarettes but no longer smoking.  Current smokers: smoking more than 100 cigarettes and currently smoking. |
| Drinking status | Non-drinker: no alcohol drinking.  Low to moderate drinker: <2 drinks/day for males and <1 drink/day for females.  Heavy drinker: ≥2 drinks/day for males and ≥1 drink/day for females. |
| Physical activity | Physical activity is evaluated in terms of the metabolic equivalent of task (MET), taking into account the duration, frequency, and intensity of vigorous and moderate exercise during leisure time each week.  Inactive: <600 MET-min/week.  Active: ≥600 MET-min/week. |
| Hypertension | Systolic blood pressure ≥140 mmHg, diastolic blood pressure ≥90 mmHg, previously diagnosed with hypertension, or use of antihypertensive medications. |
| Diabetes | Fasting blood glucose ≥126 mg/dL, glycohemoglobin ≥6.5%, previously diagnosed with diabetes, or use of antidiabetic medications. |

**Table S2.** Basic characteristics of participants based on quartiles of HbAA.

| **Characteristic** | **HbAA** | | | | ***P* value** |
| --- | --- | --- | --- | --- | --- |
|  | **Quartile 1**  **(N=361)** | **Quartile 2**  **(N=363)** | **Quartile 3**  **(N=359)** | **Quartile 4**  **(N=360)** |  |
| **Age, years** | 40.07 (11.36) | 40.15 (11.57) | 38.11 (11.88) | 38.96 (12.09) | 0.106 |
| **Sex** |  |  |  |  | 0.313 |
| Male | 176 (50.20) | 169 (47.32) | 170 (51.30) | 212 (55.25) |  |
| Female | 185 (49.80) | 194 (52.68) | 189 (48.70) | 148 (44.75) |  |
| **Race** |  |  |  |  | 0.005 |
| Non-Hispanic White | 121 (61.88) | 122 (61.94) | 129 (61.45) | 181 (68.58) |  |
| Non-Hispanic Black | 63 (10.75) | 52 (7.83) | 69 (11.92) | 91 (14.17) |  |
| Hispanic | 99 (16.63) | 132 (21.96) | 96 (16.51) | 56 (10.77) |  |
| Other | 78 (10.75) | 57 (8.27) | 65 (10.12) | 32 (6.47) |  |
| **Educational attainment** |  |  |  |  | <0.001 |
| Less than high school | 55 (9.23) | 60 (10.12) | 59 (11.70) | 89 (21.09) |  |
| High school graduate or equivalent | 62 (15.99) | 65 (18.26) | 62 (15.53) | 119 (32.84) |  |
| Some college or associated degree | 122 (36.18) | 119 (34.17) | 110 (32.53) | 114 (34.91) |  |
| College graduate or above | 122 (38.60) | 119 (37.46) | 128 (40.24) | 38 (11.16) |  |
| **Marital status** |  |  |  |  | 0.025 |
| Single | 142 (38.86) | 116 (30.70) | 137 (36.36) | 172 (44.84) |  |
| Couple | 219 (61.14) | 247 (69.30) | 222 (63.64) | 188 (55.16) |  |
| **Poverty income ratio** |  |  |  |  | <0.001 |
| <1.0 | 68 (12.86) | 58 (10.75) | 63 (13.75) | 102 (21.35) |  |
| 1.0-3.0 | 138 (35.13) | 148 (34.46) | 134 (30.41) | 176 (45.73) |  |
| ˃3.0 | 155 (52.01) | 157 (54.79) | 162 (55.84) | 82 (32.92) |  |
| **Smoking status** |  |  |  |  | <0.001 |
| Non-smoker | 288 (81.98) | 280 (75.43) | 237 (63.29) | 45 (10.90) |  |
| Former smoker | 72 (17.78) | 65 (18.93) | 66 (21.70) | 40 (15.16) |  |
| Current smoker | 1 (0.23) | 18 (5.64) | 56 (15.01) | 275 (73.95) |  |
| **Drinking status** |  |  |  |  | 0.005 |
| Non-drinker | 112 (25.42) | 105 (22.47) | 97 (17.86) | 51 (13.13) |  |
| Low to moderate | 236 (69.48) | 235 (69.81) | 235 (71.20) | 255 (70.81) |  |
| Heavy | 13 (5.10) | 23 (7.73) | 27 (10.94) | 54 (16.06) |  |
| **Physical activity** |  |  |  |  | 0.017 |
| Inactive | 215 (59.31) | 205 (55.85) | 198 (49.50) | 242 (63.63) |  |
| Active | 146 (40.69) | 158 (44.15) | 161 (50.50) | 118 (36.37) |  |
| **Hypertension** |  |  |  |  | 0.109 |
| No | 264 (74.97) | 277 (76.93) | 269 (76.16) | 245 (67.08) |  |
| Yes | 97 (25.03) | 86 (23.07) | 90 (23.84) | 115 (32.92) |  |
| **Diabetes** |  |  |  |  | 0.444 |
| No | 325 (91.73) | 327 (91.62) | 328 (94.88) | 330 (92.30) |  |
| Yes | 36 (8.27) | 36 (8.38) | 31 (5.12) | 30 (7.70) |  |

The numbers of participants in each category are unweighted observed frequencies, while means, standard errors, and percentages are population-weighted. HbAA, hemoglobin adducts of acrylamide.

**Table S3.** Basic characteristics of participants based on quartiles of HbGA.

| **Characteristic** | **HbGA** | | | | ***P* value** |
| --- | --- | --- | --- | --- | --- |
|  | **Quartile 1**  **(N=363)** | **Quartile 2**  **(N=361)** | **Quartile 3**  **(N=360)** | **Quartile 4**  **(N=359)** |  |
| **Age, years** | 39.12 (11.37) | 41.03 (11.71) | 38.53 (12.00) | 38.47 (11.76) | 0.025 |
| **Sex** |  |  |  |  | 0.787 |
| Male | 202 (53.26) | 164 (49.66) | 176 (51.63) | 185 (49.06) |  |
| Female | 161 (46.74) | 197 (50.34) | 184 (48.37) | 174 (50.94) |  |
| **Race** |  |  |  |  | 0.148 |
| Non-Hispanic White | 123 (62.40) | 128 (63.84) | 126 (59.13) | 176 (67.97) |  |
| Non-Hispanic Black | 79 (12.94) | 63 (9.69) | 59 (10.17) | 74 (11.48) |  |
| Hispanic | 87 (15.39) | 105 (15.98) | 118 (21.97) | 73 (13.57) |  |
| Other | 74 (9.26) | 65 (10.48) | 57 (8.73) | 36 (6.99) |  |
| **Educational attainment** |  |  |  |  | <0.001 |
| Less than high school | 54 (9.70) | 54 (8.24) | 69 (14.40) | 86 (19.97) |  |
| High school graduate or equivalent | 64 (16.49) | 63 (18.78) | 66 (16.46) | 115 (30.38) |  |
| Some college or associated degree | 112 (31.47) | 122 (34.67) | 120 (37.91) | 111 (33.91) |  |
| College graduate or above | 133 (42.35) | 122 (38.32) | 105 (31.23) | 47 (15.75) |  |
| **Marital status** |  |  |  |  | 0.072 |
| Single | 134 (35.60) | 139 (36.87) | 123 (32.41) | 171 (44.94) |  |
| Couple | 229 (64.40) | 222 (63.13) | 237 (67.59) | 188 (55.06) |  |
| **Poverty income ratio** |  |  |  |  | 0.011 |
| <1.0 | 68 (12.87) | 59 (11.22) | 64 (14.68) | 100 (19.79) |  |
| 1.0-3.0 | 138 (33.81) | 140 (34.13) | 149 (34.88) | 169 (42.61) |  |
| ˃3.0 | 157 (53.32) | 162 (54.64) | 147 (50.45) | 90 (37.60) |  |
| **Smoking status** |  |  |  |  | <0.001 |
| Non-smoker | 281 (79.22) | 263 (70.17) | 216 (60.41) | 90 (23.05) |  |
| Former smoker | 66 (15.87) | 72 (23.08) | 71 (20.57) | 34 (13.90) |  |
| Current smoker | 16 (4.92) | 26 (6.75) | 73 (19.02) | 235 (63.05) |  |
| **Drinking status** |  |  |  |  | 0.302 |
| Non-drinker | 107 (22.82) | 93 (20.93) | 92 (18.22) | 73 (17.07) |  |
| Low to moderate | 228 (69.32) | 239 (67.51) | 246 (74.00) | 248 (70.93) |  |
| Heavy | 28 (7.86) | 29 (11.56) | 22 (7.77) | 38 (12.00) |  |
| **Physical activity** |  |  |  |  | 0.099 |
| Inactive | 196 (55.19) | 211 (56.00) | 210 (53.03) | 243 (63.69) |  |
| Active | 167 (44.81) | 150 (44.00) | 150 (46.97) | 116 (36.31) |  |
| **Hypertension** |  |  |  |  | 0.264 |
| No | 272 (75.96) | 265 (75.75) | 266 (74.92) | 252 (68.80) |  |
| Yes | 91 (24.04) | 96 (24.25) | 94 (25.08) | 107 (31.20) |  |
| **Diabetes** |  |  |  |  | 0.425 |
| No | 336 (94.63) | 334 (93.10) | 318 (91.28) | 322 (91.26) |  |
| Yes | 27 (5.37) | 27 (6.90) | 42 (8.72) | 37 (8.74) |  |

The numbers of participants in each category are unweighted observed frequencies, while means, standard errors, and percentages are population-weighted. HbGA, hemoglobin adducts of glycidamide.

**Table S4.** Basic characteristics of participants based on quartiles of HbGA/HbAA ratio.

| **Characteristic** | **HbGA/HbAA ratio** | | | | ***P* value** |
| --- | --- | --- | --- | --- | --- |
|  | **Quartile 1**  **(N=361)** | **Quartile 2**  **(N=361)** | **Quartile 3**  **(N=360)** | **Quartile 4**  **(N=361)** |  |
| **Age, years** | 38.61 (12.46) | 39.91 (11.46) | 38.86 (11.66) | 39.95 (11.39) | 0.345 |
| **Sex** |  |  |  |  | <0.001 |
| Male | 228 (61.16) | 196 (53.56) | 169 (50.74) | 134 (38.83) |  |
| Female | 133 (38.84) | 165 (46.44) | 191 (49.26) | 227 (61.17) |  |
| **Race** |  |  |  |  | <0.001 |
| Non-Hispanic White | 139 (61.27) | 141 (65.33) | 145 (63.83) | 128 (62.87) |  |
| Non-Hispanic Black | 117 (19.31) | 70 (11.05) | 53 (8.50) | 35 (6.05) |  |
| Hispanic | 50 (9.97) | 94 (15.83) | 100 (16.81) | 139 (23.52) |  |
| Other | 55 (9.45) | 56 (7.79) | 62 (10.87) | 59 (7.56) |  |
| **Educational attainment** |  |  |  |  | 0.243 |
| Less than high school | 74 (14.77) | 66 (13.34) | 57 (11.51) | 66 (12.05) |  |
| High school graduate or equivalent | 89 (23.85) | 71 (18.71) | 78 (21.60) | 70 (17.86) |  |
| Some college or associated degree | 113 (32.68) | 103 (30.15) | 126 (38.25) | 123 (36.44) |  |
| College graduate or above | 85 (28.70) | 121 (37.81) | 99 (28.64) | 102 (33.65) |  |
| **Marital status** |  |  |  |  | 0.278 |
| Single | 150 (38.25) | 145 (37.52) | 149 (41.64) | 123 (32.32) |  |
| Couple | 211 (61.75) | 216 (62.48) | 211 (58.36) | 238 (67.68) |  |
| **Poverty income ratio** |  |  |  |  | 0.106 |
| <1.0 | 82 (16.09) | 81 (17.69) | 60 (11.99) | 68 (12.43) |  |
| 1.0-3.0 | 157 (38.54) | 139 (33.61) | 165 (40.77) | 135 (32.19) |  |
| ˃3.0 | 122 (45.37) | 141 (48.70) | 135 (47.23) | 158 (55.37) |  |
| **Smoking status** |  |  |  |  | <0.001 |
| Non-smoker | 126 (35.57) | 219 (61.01) | 234 (63.91) | 271 (73.02) |  |
| Former smoker | 59 (19.03) | 54 (16.53) | 68 (19.92) | 62 (18.34) |  |
| Current smoker | 176 (45.40) | 88 (22.46) | 58 (16.17) | 28 (8.65) |  |
| **Drinking status** |  |  |  |  | <0.001 |
| Non-drinker | 62 (11.83) | 84 (19.50) | 81 (16.68) | 138 (30.90) |  |
| Low to moderate | 233 (68.68) | 256 (72.42) | 256 (75.11) | 216 (64.76) |  |
| Heavy | 66 (19.49) | 21 (8.08) | 23 (8.20) | 7 (4.34) |  |
| **Physical activity** |  |  |  |  | 0.030 |
| Inactive | 213 (56.60) | 206 (50.80) | 212 (56.33) | 229 (64.11) |  |
| Active | 148 (43.40) | 155 (49.20) | 148 (43.67) | 132 (35.89) |  |
| **Hypertension** |  |  |  |  | 0.573 |
| No | 261 (71.68) | 258 (73.72) | 272 (76.87) | 264 (73.22) |  |
| Yes | 100 (28.32) | 103 (26.28) | 88 (23.13) | 97 (26.78) |  |
| **Diabetes** |  |  |  |  | 0.003 |
| No | 342 (96.59) | 336 (94.36) | 328 (93.20) | 304 (86.59) |  |
| Yes | 19 (3.41) | 25 (5.64) | 32 (6.80) | 57 (13.41) |  |

The numbers of participants in each category are unweighted observed frequencies, while means, standard errors, and percentages are population-weighted. HbGA, hemoglobin adducts of glycidamide; HbAA, hemoglobin adducts of acrylamide.

**Table S5.** Mediation effects of inflammation and oxidant stress in the association of acrylamide with muscle mass index among U.S. adults in NHANES 2013-2016.

| **Mediator** | **ACME** | **ADE** | **Total effect** | **Prop. mediated** | ***P* value** |
| --- | --- | --- | --- | --- | --- |
| **HbAA** |  |  |  |  |  |
| WBC | 0.0350  (0.0008, 0.0678) | -0.3920  (-0.5365, -0.2532) | -0.3570  (-0.5032, -0.2202) | 9.81% | 0.049 |
| NEU | 0.0322  (0.0061, 0.0604) | -0.3892  (-0.5362, -0.2489) | -0.3570  (-0.5084, -0.2145) | 9.03% | 0.014 |
| LYM | 0.0029  (-0.0260, 0.0316) | -0.3598  (-0.5119, -0.2141) | -0.3570  (-0.5075, -0.1992) | NA | 0.870 |
| UA | -0.1120  (-0.1571, -0.0730) | -0.2449  (-0.4059, -0.1008) | -0.3570  (-0.5242, -0.2140) | 31.39% | <0.001 |
| GGT | 0.0010  (-0.0088, 0.0048) | -0.3580  (-0.5106, -0.2112) | -0.3570  (-0.5101, -0.2145) | NA | 0.884 |
| TB | 0.0103  (-0.0072, 0.0297) | -0.3672  (-0.5080, -0.2237) | -0.3570  (-0.5004, -0.2109) | NA | 0.270 |
| **HbGA/HbAA** **ratio** |  |  |  |  |  |
| WBC | 0.1282  (0.0778, 0.1902) | 0.7907  (0.5295, 1.0654) | 0.9190  (0.6569, 1.1984) | 13.95% | <0.001 |
| NEU | 0.0797  (0.0409, 0.1294) | 0.8392  (0.5570, 1.1182) | 0.9190  (0.6377, 1.2002) | 8.68% | <0.001 |
| LYM | 0.0941  (0.0442, 0.1519) | 0.8249  (0.5513, 1.1174) | 0.9190  (0.6473, 1.2185) | 10.24% | <0.001 |
| UA | 0.1147  (0.0399, 0.1954) | 0.8043  (0.5581, 1.0870) | 0.9190  (0.6631, 1.2068) | 12.48% | <0.001 |
| GGT | -0.0039  (-0.0307, 0.0163) | 0.9229  (0.6801, 1.2031) | 0.9190  (0.6800, 1.1905) | NA | 0.686 |
| TB | 0.0545  (0.0202, 0.0998) | 0.8644  (0.6125, 1.1495) | 0.9190  (0.6712, 1.2176) | 5.93% | <0.001 |

ACME, average causal mediation effects; ADE, average direct effects; HbAA, hemoglobin adducts of acrylamide; HbGA, hemoglobin adducts of glycidamide; WBC, white blood cell; NEU, neutrophil; LYM, Lymphocyte; UA, uric acid; GGT, gamma-glutamyl transferase; TB, total bilirubin.

**Table S6.** Stratified analysis of the weighted association between HbAA and muscle mass index among U.S. adults in NHANES 2013-2016.

| **Characteristic** | ***β* (95% CI)** | ***P* for interaction** |
| --- | --- | --- |
| **Age** |  | 0.082 |
| 20-39 years | -0.469 (-0.665, -0.274) |  |
| 40-59 years | -0.132 (-0.421, 0.157) |  |
| **Sex** |  | 0.598 |
| Male | -0.208 (-0.419, 0.003) |  |
| Female | -0.528 (-0.816, -0.239) |  |
| **Race** |  | 0.303 |
| Non-Hispanic White | -0.304 (-0.559, -0.048) |  |
| Non-Hispanic Black | -0.171 (-0.434, 0.092) |  |
| Hispanic | -0.449 (-0.850, -0.048) |  |
| Other | -0.166 (-0.641, 0.310) |  |
| **Educational attainment** |  | 0.410 |
| Less than high school | -0.516 (-0.817, -0.215) |  |
| High school graduate or equivalent | -0.183 (-0.490, 0.125) |  |
| Some college or associated degree | -0.351 (-0.616, -0.087) |  |
| College graduate or above | -0.300 (-0.743, 0.142) |  |
| **Marital status** |  | 0.349 |
| Single | -0.533 (-0.774, -0.292) |  |
| Couple | -0.201 (-0.475, 0.073) |  |
| **Poverty income ratio** |  | 0.056 |
| <1.0 | -0.460 (-0.862, -0.058) |  |
| 1.0-3.0 | -0.476 (-0.683, -0.270) |  |
| ˃3.0 | -0.143 (-0.405, 0.118) |  |
| **Smoking status** |  | 0.052 |
| Non-smoker | -0.446 (-0.660, -0.231) |  |
| Former smoker | 0.104 (-0.285, 0.493) |  |
| Current smoker | -0.529 (-0.745, -0.313) |  |
| **Drinking status** |  | 0.258 |
| Non-drinker | -0.157 (-0.539, 0.225) |  |
| Low to moderate | -0.418 (-0.634, -0.201) |  |
| Heavy | -0.270 (-0.696, 0.156) |  |
| **Physical activity** |  | 0.560 |
| Inactive | -0.382 (-0.693, -0.070) |  |
| Active | -0.266 (-0.459, -0.074) |  |
| **Hypertension** |  | 0.194 |
| No | -0.288 (-0.465, -0.112) |  |
| Yes | -0.442 (-0.891, 0.007) |  |
| **Diabetes** |  | 0.740 |
| No | -0.326 (-0.524, -0.129) |  |
| Yes | -0.423 (-0.831, -0.014) |  |

The models were adjusted for age, sex, race, educational level, marital status, poverty income ratio, smoking status, drinking status, physical activity, hypertension, and diabetes, except for the corresponding stratification variable. Blood acrylamide was natural logarithm transformed before analyses. HbAA, hemoglobin adducts of acrylamide; CI, confidence interval.

**Table S7.** Stratified analysis of the weighted association between HbGA/HbAA ratio and muscle mass index among U.S. adults in NHANES 2013-2016.

| **Characteristic** | ***β* (95% CI)** | ***P* for interaction** |
| --- | --- | --- |
| **Age** |  | 0.553 |
| 20-39 years | 0.960 (0.596, 1.324) |  |
| 40-59 years | 1.137 (0.696, 1.578) |  |
| **Sex** |  | 0.648 |
| Male | 0.971 (0.585, 1.358) |  |
| Female | 1.099 (0.694, 1.504) |  |
| **Race** |  | 0.138 |
| Non-Hispanic White | 1.147 (0.829, 1.464) |  |
| Non-Hispanic Black | 0.914 (0.263, 1.566) |  |
| Hispanic | 0.583 (-0.006, 1.172) |  |
| Other | 0.834 (0.175, 1.492) |  |
| **Educational attainment** |  | 0.704 |
| Less than high school | 0.876 (0.369, 1.383) |  |
| High school graduate or equivalent | 0.938 (0.420, 1.456) |  |
| Some college or associated degree | 1.108 (0.536, 1.679) |  |
| College graduate or above | 0.956 (0.495, 1.417) |  |
| **Marital status** |  | 0.108 |
| Single | 1.314 (0.908, 1.721) |  |
| Couple | 0.874 (0.533, 1.214) |  |
| **Poverty income ratio** |  | 0.864 |
| <1.0 | 1.112 (0.364, 1.860) |  |
| 1.0-3.0 | 0.949 (0.572, 1.326) |  |
| ˃3.0 | 1.071 (0.705, 1.438) |  |
| **Smoking status** |  | 0.573 |
| Non-smoker | 0.876 (0.450, 1.301) |  |
| Former smoker | 1.099 (0.441, 1.757) |  |
| Current smoker | 1.220 (0.792, 1.648) |  |
| **Drinking status** |  | 0.600 |
| Non-drinker | 1.486 (0.709, 2.264) |  |
| Low to moderate | 0.943 (0.654, 1.232) |  |
| Heavy | 1.171 (0.651, 1.691) |  |
| **Physical activity** |  | 0.028 |
| Inactive | 1.311 (0.982, 1.641) |  |
| Active | 0.651 (0.234, 1.068) |  |
| **Hypertension** |  | 0.417 |
| No | 0.962 (0.655, 1.270) |  |
| Yes | 1.219 (0.652, 1.786) |  |
| **Diabetes** |  | 0.670 |
| No | 1.031 (0.721, 1.340) |  |
| Yes | 1.224 (0.576, 1.872) |  |

The models were adjusted for age, sex, race, educational level, marital status, poverty income ratio, smoking status, drinking status, physical activity, hypertension, and diabetes, except for the corresponding stratification variable. Blood acrylamide was natural logarithm transformed before analyses. HbAA, hemoglobin adducts of acrylamide; HbGA, hemoglobin adducts of glycidamide; CI, confidence interval.

**Table S8.** Association between acrylamide and muscle mass index among U.S. adults in NHANES 2013-2016, after applying multiple imputations for missing covariates.

| **Variable** | ***β* (95% CI)** | ***P* value** |
| --- | --- | --- |
| HbAA |  |  |
| Per unit increase | **-0.298 (-0.504, -0.093)** | **0.008** |
| Quartile 1 | Reference |  |
| Quartile 2 | -0.263 (-0.599, 0.074) | 0.111 |
| Quartile 3 | -0.214 (-0.469, 0.040) | 0.089 |
| Quartile 4 | **-0.420 (-0.801, -0.039)** | **0.034** |
| HbGA |  |  |
| Per unit increase | 0.013 (-0.190, 0.216) | 0.892 |
| Quartile 1 | Reference |  |
| Quartile 2 | 0.182 (-0.061, 0.424) | 0.124 |
| Quartile 3 | 0.107 (-0.147, 0.361) | 0.365 |
| Quartile 4 | 0.124 (-0.195, 0.443) | 0.401 |
| HbGA/HbAA ratio |  |  |
| Per unit increase | **0.875 (0.534, 1.215)** | **<0.001** |
| Quartile 1 | Reference |  |
| Quartile 2 | 0.178 (-0.059, 0.416) | 0.124 |
| Quartile 3 | **0.368 (0.057, 0.679)** | **0.025** |
| Quartile 4 | **0.751 (0.475, 1.027)** | **<0.001** |

The models were adjusted for age, sex, race, educational level, marital status, poverty income ratio, smoking status, drinking status, physical activity, hypertension, and diabetes. Blood acrylamide was natural logarithm transformed before analyses. Results in bold indicate statistical significance. HbAA, hemoglobin adducts of acrylamide; HbGA, hemoglobin adducts of glycidamide; CI, confidence interval.

**Table S9.** Association between acrylamide and muscle mass index among U.S. adults in NHANES 2013-2016, after excluding those who had a history of cancer.

| **Variable** | ***β* (95% CI)** | ***P* value** |
| --- | --- | --- |
| HbAA |  |  |
| Per unit increase | **-0.366 (-0.556, -0.175)** | **0.001** |
| Quartile 1 | Reference |  |
| Quartile 2 | -0.301 (-0.695, 0.093) | 0.118 |
| Quartile 3 | -0.259 (-0.554, 0.036) | 0.078 |
| Quartile 4 | **-0.541 (-0.906, -0.176)** | **0.008** |
| HbGA |  |  |
| Per unit increase | -0.016 (-0.225, 0.193) | 0.868 |
| Quartile 1 | Reference |  |
| Quartile 2 | -0.001 (-0.239, 0.238) | 0.995 |
| Quartile 3 | 0.081 (-0.196, 0.358) | 0.523 |
| Quartile 4 | 0.052 (-0.298, 0.401) | 0.747 |
| HbGA/HbAA ratio |  |  |
| Per unit increase | **1.037 (0.698, 1.376)** | **<0.001** |
| Quartile 1 | Reference |  |
| Quartile 2 | 0.169 (-0.12, 0.459) | 0.218 |
| Quartile 3 | **0.385 (0.032, 0.737)** | **0.036** |
| Quartile 4 | **0.806 (0.508, 1.103)** | **<0.001** |

The models were adjusted for age, sex, race, educational level, marital status, poverty income ratio, smoking status, drinking status, physical activity, hypertension, and diabetes. Blood acrylamide was natural logarithm transformed before analyses. Results in bold indicate statistical significance. HbAA, hemoglobin adducts of acrylamide; HbGA, hemoglobin adducts of glycidamide; CI, confidence interval.

**Table S10.** Association between acrylamide and muscle mass index among U.S. adults in NHANES 2013-2016, after excluding participants identified as outliers in their exposure values.

| **Variable** | ***β* (95% CI)** | ***P* value** |
| --- | --- | --- |
| HbAA |  |  |
| Per unit increase | **-0.410 (-0.617, -0.202)** | **0.001** |
| Quartile 1 | Reference |  |
| Quartile 2 | -0.268 (-0.653, 0.116) | 0.149 |
| Quartile 3 | **-0.262 (-0.516, -0.008)** | **0.045** |
| Quartile 4 | **-0.438 (-0.806, -0.071)** | **0.024** |
| HbGA |  |  |
| Per unit increase | 0.054 (-0.156, 0.264) | 0.580 |
| Quartile 1 | Reference |  |
| Quartile 2 | 0.099 (-0.116, 0.315) | 0.324 |
| Quartile 3 | 0.058 (-0.190, 0.307) | 0.610 |
| Quartile 4 | 0.084 (-0.228, 0.397) | 0.556 |
| HbGA/HbAA ratio |  |  |
| Per unit increase | **1.058 (0.752, 1.363)** | **<0.001** |
| Quartile 1 | Reference |  |
| Quartile 2 | 0.186 (-0.100, 0.471) | 0.175 |
| Quartile 3 | **0.358 (0.002, 0.715)** | **0.049** |
| Quartile 4 | **0.765 (0.522, 1.009)** | **<0.001** |

The models were adjusted for age, sex, race, educational level, marital status, poverty income ratio, smoking status, drinking status, physical activity, hypertension, and diabetes. Blood acrylamide was natural logarithm transformed before analyses. Results in bold indicate statistical significance. HbAA, hemoglobin adducts of acrylamide; HbGA, hemoglobin adducts of glycidamide; CI, confidence interval.

**Table S11.** Association between acrylamide and muscle mass index among U.S. adults in NHANES 2013-2016, using unweighted data.

| **Variable** | ***β* (95% CI)** | ***P* value** |
| --- | --- | --- |
| HbAA |  |  |
| Per unit increase | **-0.357 (-0.502, -0.212)** | **<0.001** |
| Quartile 1 | Reference |  |
| Quartile 2 | **-0.236 (-0.431, -0.040)** | **0.018** |
| Quartile 3 | **-0.217 (-0.416, -0.019)** | **0.032** |
| Quartile 4 | **-0.487 (-0.752, -0.222)** | **<0.001** |
| HbGA |  |  |
| Per unit increase | -0.045 (-0.191, 0.101) | 0.548 |
| Quartile 1 | Reference |  |
| Quartile 2 | 0.098 (-0.098, 0.294) | 0.328 |
| Quartile 3 | 0.026 (-0.174, 0.226) | 0.800 |
| Quartile 4 | -0.003 (-0.237, 0.230) | 0.977 |
| HbGA/HbAA ratio |  |  |
| Per unit increase | **0.919 (0.674, 1.164)** | **<0.001** |
| Quartile 1 | Reference |  |
| Quartile 2 | **0.320 (0.118, 0.521)** | **0.002** |
| Quartile 3 | **0.436 (0.229, 0.642)** | **<0.001** |
| Quartile 4 | **0.731 (0.513, 0.950)** | **<0.001** |

The models were adjusted for age, sex, race, educational level, marital status, poverty income ratio, smoking status, drinking status, physical activity, hypertension, and diabetes. Blood acrylamide was natural logarithm transformed before analyses. Results in bold indicate statistical significance. HbAA, hemoglobin adducts of acrylamide; HbGA, hemoglobin adducts of glycidamide; CI, confidence interval.


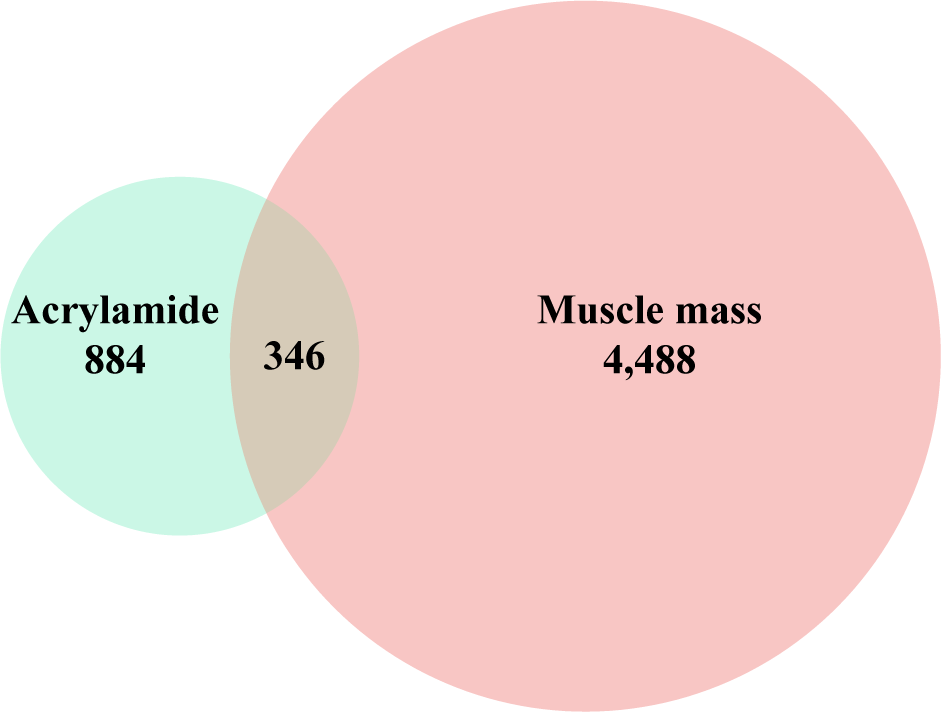


**Figure S1.** Venn graph of potential targets of acrylamide exposure and reduced muscle mass.
